# IgG Responses to Pneumococcal and Haemophilus Influenzae Protein Antigens Are Not Impaired in Children with a History of Recurrent Acute Otitis Media

**DOI:** 10.1371/journal.pone.0049061

**Published:** 2012-11-12

**Authors:** Selma P. Wiertsema, Karli J. Corscadden, Eva N. Mowe, Guicheng Zhang, Shyan Vijayasekaran, Harvey L. Coates, Timothy J. Mitchell, Wayne R. Thomas, Peter C. Richmond, Lea-Ann S. Kirkham

**Affiliations:** 1 School of Paediatrics and Child Health, University of Western Australia, Perth, Australia; 2 Centre for Child Health Research, Telethon Institute for Child Health Research, Perth, Australia; 3 Department of Otolaryngology, Head and Neck Surgery, University of Western Australia, Perth, Australia; 4 Division of Infection and Immunity, University of Glasgow, Glasgow, United Kingdom; Instituto Butantan, Brazil

## Abstract

**Background:**

Vaccines including conserved antigens from *Streptococcus pneumoniae* and nontypeable *Haemophilus influenzae* (NTHi) have the potential to reduce the burden of acute otitis media. Little is known about the antibody response to such antigens in young children with recurrent acute otitis media, however, it has been suggested antibody production may be impaired in these children.

**Methods:**

We measured serum IgG levels against 4 pneumococcal (PspA1, PspA 2, CbpA and Ply) and 3 NTHi (P4, P6 and PD) proteins in a cross-sectional study of 172 children under 3 years of age with a history of recurrent acute otitis media (median 7 episodes, requiring ventilation tube insertion) and 63 healthy age-matched controls, using a newly developed multiplex bead assay.

**Results:**

Children with a history of recurrent acute otitis media had significantly higher geometric mean serum IgG levels against NTHi proteins P4, P6 and PD compared with healthy controls, whereas there was no difference in antibody levels against pneumococcal protein antigens. In both children with and without a history of acute otitis media, antibody levels increased with age and were significantly higher in children colonised with *S. pneumoniae* or NTHi compared with children that were not colonised.

**Conclusions:**

Proteins from *S. pneumoniae* and NTHi induce serum IgG in children with a history of acute otitis media. The mechanisms in which proteins induce immunity and potential protection requires further investigation but the dogma of impaired antibody responses in children with recurrent acute otitis media should be reconsidered.

## Introduction

Acute otitis media (AOM) is the most common reason for physician visits and the prescription of antibiotics for children in industrialised countries, where 80% of children will have an episode of AOM at least once before age 3 and infants spend a mean of 42 days on antibiotics in the first year of life [1]. Data from the USA suggests that the costs associated with AOM are approximately US$3.5 billion per year and the World Health Organization estimates that in developing countries 51,000 deaths per year in children younger than 5 years of age are attributable to complications of AOM [1].

*Streptococcus pneumoniae* and nontypeable *Haemophilus influenzae* (NTHi) account for approximately 80% of AOM cases and exposure to these pathogens early in life is a risk-factor for the development of recurrent AOM (rAOM) [2]. Although *S. pneumoniae* is a predominant AOM pathogen, the 7-valent pneumococcal conjugate vaccine (PCV7) has had modest impact on overall otitis media rates, which is partly due to replacement of PCV7 serotypes with non-PCV7 serotypes and other pathogens after the widespread use of PCV7 [3], [4], [5]. Vaccines that confer species-wide protection against multiple bacterial pathogens, with the potential to prevent early bacterial colonisation and subsequent rAOM, may be more successful and are urgently required. A trial with an 11-valent predecessor of the currently licensed 10-valent pneumococcal conjugate vaccine (PCV10) that included Protein D from NTHi as a carrier protein, showed that the incidence of NTHi AOM decreased by 35%, suggesting protein-based vaccines may be effective against AOM [6]. Indeed several protein antigens of *S. pneumoniae* and NTHi have been demonstrated to be protective against otitis media in animal models [7], [8], [9], however little is known about the antibody response to such antigens in young children with rAOM. The few studies investigating natural acquired humoral immunity against bacterial proteins in children with AOM are contradictory, with studies indicating that children with AOM produce antibodies against pneumococcal proteins [10], [11], [12], [13], [14], whereas others have shown impaired antibody production against pneumococcal and NTHi proteins in otitis-prone children, suggesting these children have a specific humoral immunodeficiency [15], [16], [17], [18], [19]. Group sizes in these studies are generally small, have a wide age-range and/or do not include healthy age-matched controls. In addition, antibody levels are often measured against a limited number of protein antigens due to the small blood sample volumes available from young children.

To address whether children with rAOM are able to mount antibody responses to surface exposed bacterial proteins that are potential vaccine candidates, we have measured serum IgG antibody levels to 4 pneumococcal and 3 NTHi proteins in a cross-sectional cohort of 172 children less than 3 years of age requiring the insertion of ventilation tubes for rAOM and 63 healthy age-matched controls. We developed a novel bead-based multiplex assay that permits the measurement of antibody levels to all 7 protein antigens in less than 10 µl of serum. Our study showed that humoral immunity against *S. pneumoniae* and NTHi is not impaired in children with rAOM, which are important data when developing protein-based vaccines that aim to reduce the burden of AOM.

## Methods

### Ethics Statement

The study was approved by the Ethics Committee of Princess Margaret Hospital for Children, Perth, Western Australia (1295/EP) and by the institutional boards of the private hospitals in Perth where recruitment took place. Written informed consent was obtained from parents of participating children before recruitment.

**Figure 1:**
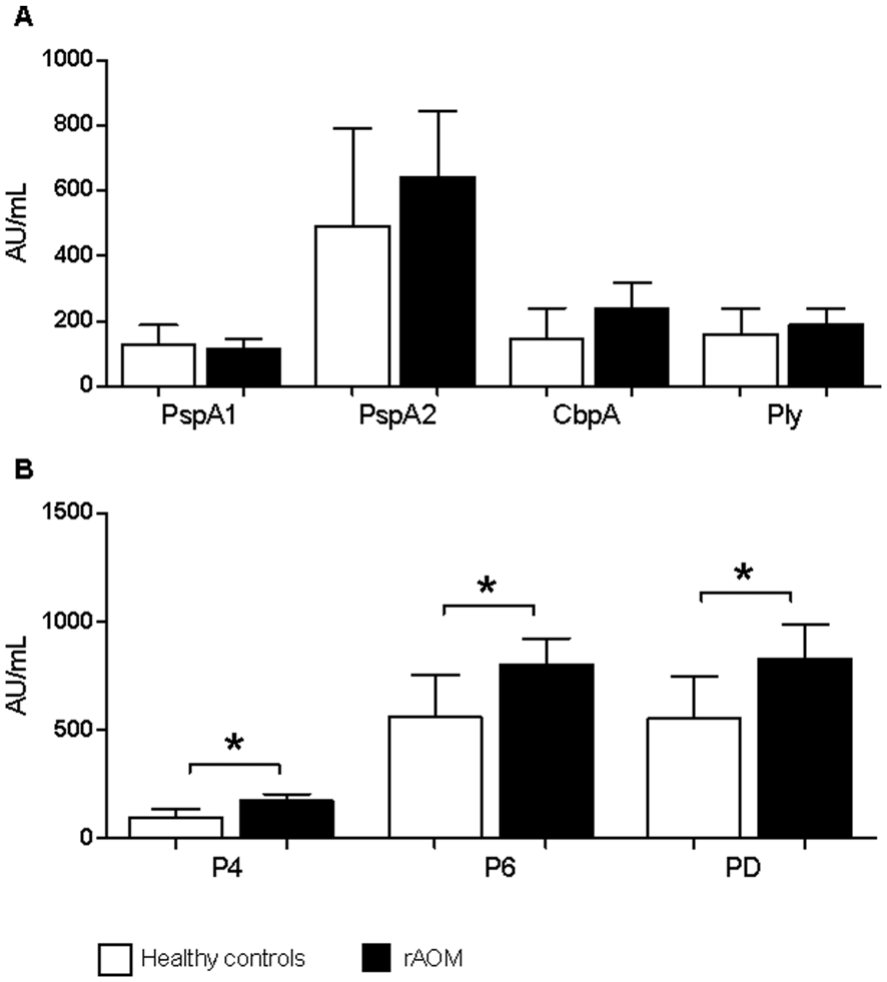
Serum IgG levels. Adjusted (age, gender, day-care attendance) geometric mean concentration of serum IgG antibody levels in Arbituary Units/mL (95% CI) against pneumococcal (top panel A) and NTHi (bottom panel B) proteins in healthy children (white bars) and children with a history of rAOM (black bars). * p≤0.01 when comparing IgG levels between children with and without a history of rAOM using linear regression correcting for age, gender and day-care attendance *PspA1/2, pneumococcal surface protein A families 1 and 2; CbpA, choline binding protein A; Ply, pneumolysin; P4, outer membrane protein 4; P6, outer membrane protein 6; PD, protein D.*

### Recruitment of the Study Cohort

From November 2007 to May 2009, children between 6 and 36 months of age were recruited from private hospitals in Perth, Western Australia, to this cross-sectional study to investigate the immunology and microbiology of rAOM. Cases were defined as children with a history of at least 3 episodes of AOM requiring insertion of ventilation tubes. The frequency of and the time-intervals between AOM episodes that the child had experienced before entry into the study at the day of surgery were based on the ENT surgeon’s medical records and parental report. In a questionnaire, data on the number of GP visits for OM, the number of antibiotic courses, whether the child had been noticeably sick with otalgia, age at first AOM episode and number of AOM episodes in the past 12 months were collected to ascertain the recurrent AOM phenotype. When the child had not experienced at least 3 AOM episodes and was having ventilation tubes inserted for otitis media with effusion only, the child was not included in the study. Children with no history of any form of ear disease based on parental questionnaire data and undergoing general surgery (orthopaedics, strabismus, circumcision, cryptorchidism, hypospadias repair) were recruited as controls. None of the children had signs of acute infection at the time of surgery when the samples were collected. Children with diagnosed immunodeficiency, cystic fibrosis, immotile cilia syndrome, craniofacial abnormalities, and chromosomal or genetic syndromes were excluded. Data on host- and environmental risk factors (age, gender, day-care attendance, siblings, and PCV7 vaccine status) were collected by parental questionnaires and from medical records. Infants in Australia received 3 doses of PCV7 with no booster in the second year of life (3+0 schedule) at the time of this study.

**Figure 2:**
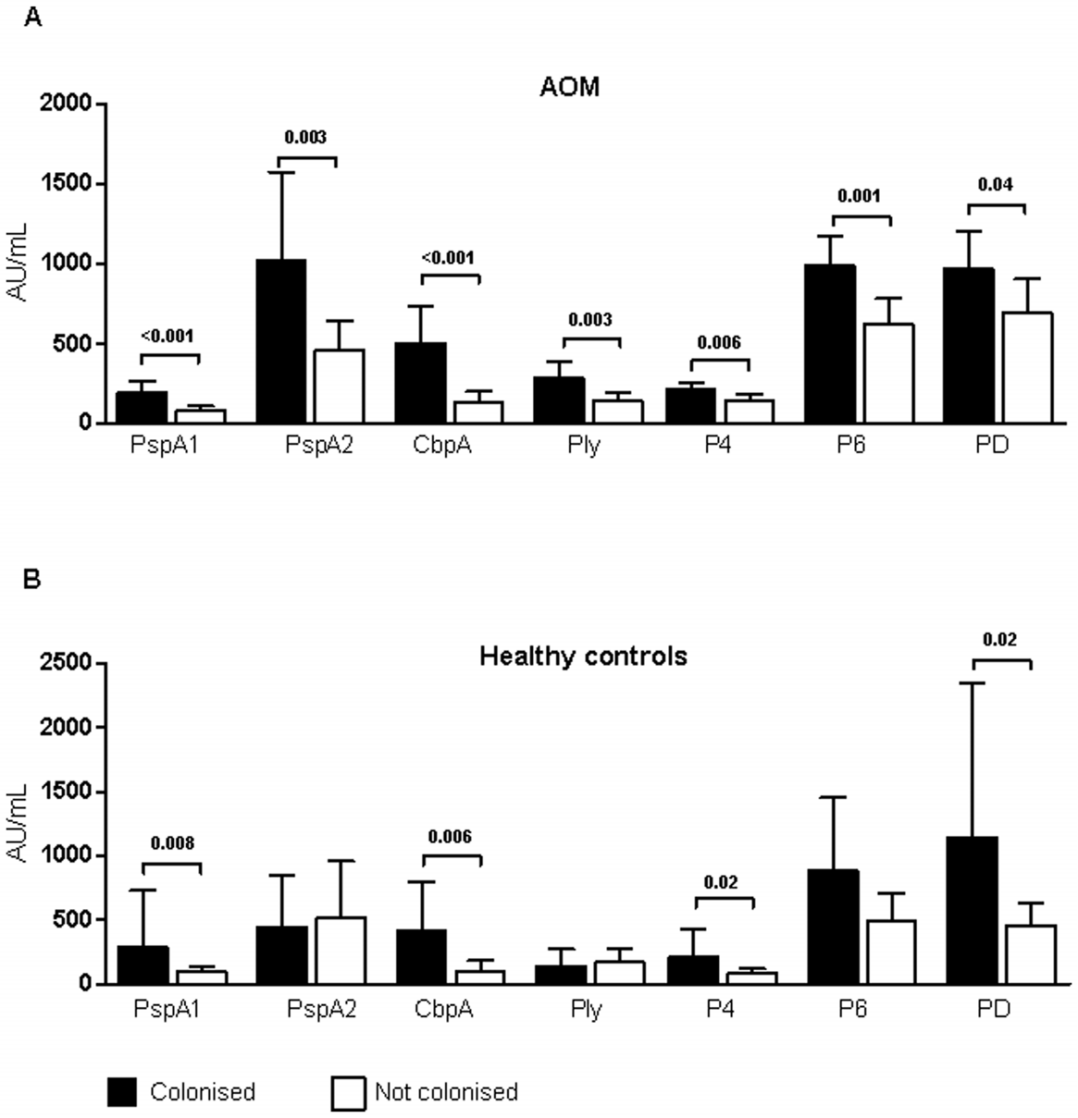
Serum IgG levels according to AOM history and nasopharyngeal colonisation. Adjusted (age, gender, day-care attendance) geometric mean concentration of serum IgG in Arbitrary Units/mL (95% CI) against pneumococcal and NTHi proteins in children positive (black bar) or negative (white bar) for nasopharyngeal colonisation with *S. pneumoniae* for IgG against PspA1, PspA2, CbpA, and Ply or nasopharyngeal colonisation with NTHi for IgG against P4, P6, and PD in children with a history of rAOM (top panel A) or healthy controls (bottom panel B). P-value determined using linear regression correcting for age, gender and day-care attendance… *PspA1/2, pneumococcal surface protein A families 1 and 2; CbpA, choline binding protein A; Ply, pneumolysin; P4, outer membrane protein 4; P6, outer membrane protein 6; PD, protein D.*

### Sample Collection

All samples were collected while the child was under general anaesthetic for surgery to insert ventilation tubes (rAOM group) or other minor surgical procedures (healthy controls). Blood samples were collected in a serum clot tube (Vacuette® Greiner Bio-one) and centrifuged for 10 min at 3200 g. The serum top-layer was collected and aliquoted. Nasopharyngeal swabs were collected by trans-nasal insertion of a sterile flexible cotton-wool swab (Copan, Brescia, Italy) reaching the nasopharyngeal space. Swabs were stored in sterile Skim-Milk-Tryptone-Glucose-Glycerol-Broth. An anterior-inferior myringotomy incision was made for the collection of middle ear effusion in 1 mL of saline with a sterile Leukotrap® (Pall Corporation, New York, USA). All samples were stored at −80°C until analyses [20].

**Table 1:** Serum IgG according to detection of *S. pneumoniae* in the middle ear.

	MEE Pnc positive(n = 8)	MEE Pnc negative(n = 126)	p-value
**PspA1**	189.6 (38.5–934.4)	126·4 (93.6–170.9)	0.5
**PspA2**	355.5 (71.8–1760.2)	784·3 (575.3–1069.4)	0.2
**CbpA**	258.6 (31.0–2161.8)	256·6 (180.8–364.1)	1.0
**Ply**	165.5 (21.5–1273.5)	199·9 (148.0–269.9)	0.8

Geometric mean concentration of serum IgG in Arbitrary Units/mL (95% CI) against pneumococcal proteins in children with a history of rAOM who do or do not have *S. pneumoniae* (Pnc) detected in middle ear effusion (MEE) using PCR. P-value determined using Students *t* test.

*PspA1/2, pneumococcal surface protein A families 1 and 2; CbpA, choline binding protein A; Ply, pneumolysin.*

**Table 2:** Serum IgG according to detection of NTHi in the middle ear.

	MEE NTHi positive(n = 61)	MEE NTHi negative(n = 73)	p-value
**P4**	195.1 (149.9–254.1)	167.0 (133.5–208.9)	0.4
**P6**	923.2 (731.1–1165.9)	797.1 (641.8–990.1)	0.4
**PD**	964.7 (748.4–1243.4)	789.8 (587.0–1062.7)	0.3

Geometric mean concentration of serum IgG in Arbitrary Units/mL (95% CI) against NTHi proteins in children with a history of rAOM who do or do not have NTHi detected in middle ear effusion (MEE) using PCR. P-value determined using Students *t* test.

*P4, outer membrane protein 4; P6, outer membrane protein 6; PD, protein D.*

### Protein Preparation

*S. pneumoniae* antigens pneumococcal surface protein A family 1 and 2 (PspA1 and PspA2) and choline binding protein A (CbpA) were constructed with C-terminal hexahistidine tags and without their repetitive choline binding sequences. Recombinant PspA1 was constituted by the 302 N-terminal amino acids of the mature PspA antigen of *S. pneumoniae* strain Rx1, and PspA2 by the 410 N-terminal amino acids of the mature PspA of *S. pneumoniae* Taiwan19F-14. Recombinant CbpA was comprised of amino acids 1 to 445 from *S. pneumoniae* strain L81905. Protein 4 (P4) and Protein 6 (P6) proteins were constructed from *H. influenzae* Eagen sequences and incorporating an N-terminal hexahistidine fusion tag [21]. Recombinant PspA1, PspA2, CbpA, P4, and P6 were expressed in *Escherichia coli* BL21 Star™(DE3)pLysS (Invitrogen, Victoria, Australia) and purified from the soluble lysate by Ni-NTA in the presence of 600 mM NaCl. Protein-containing fractions were dialysed in PBS and further purified by Bio-Rad Macro-prep High Q anion exchange chromatography (Bio-Rad, California, USA). These proteins were then purified by size exclusion over a HiPrep HR S200 26/60 column and endotoxin was removed using 0.2-µm Mustang E filters (Pall Life Sciences, Portsmouth, UK). Pneumolysin (Ply) from *S. pneumoniae* serotype 1 sequence type 306 was purified as previously described [22]. Protein purity was checked by sodium dodecyl sulphate-polyacrylamide gel electrophoresis. Non-lipidated Protein D (PD) was kindly provided by GlaxoSmithKline (GlaxoSmithKline Biologicals, Rixensart, Belgium). All proteins were stored in aliquots at −80°C.

**Figure 3:**
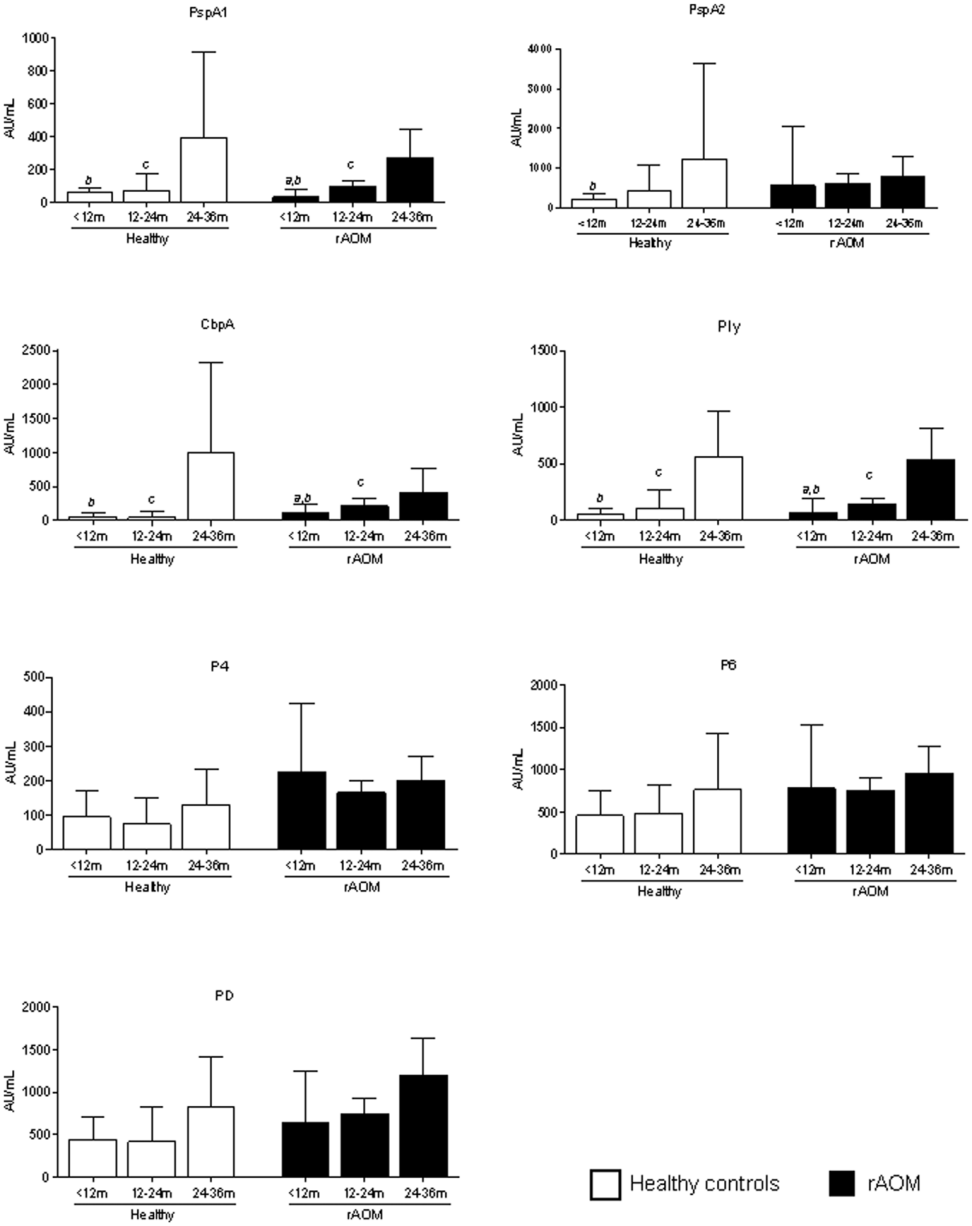
Serum IgG levels according to AOM history and age. Adjusted (gender, day-care attendance) geometric mean concentration according to age of serum IgG in Arbitrary Units/mL (95% CI) against pneumococcal and NTHi protein antigens in healthy children (white bars) and children with a history of rAOM (black bars).General linear model followed by Tukey honestly significant difference test was used to compare differences between age groups within the group of children with rAOM and within the healthy control group, where p<0.05 was considered significant; *a*  =  p<0.05 when comparing <12 months with 12–14 months, *b*  =  p<0.05 when comparing <12 months with 24–36 months, *c*  =  p<0.05 when comparing 12–24 months with 24–36 months. *PspA1/2, pneumococcal surface protein A families 1 and 2; CbpA, choline binding protein A; Ply, pneumolysin; P4, outer membrane protein 4; P6, outer membrane protein 6; PD, protein D.*

**Figure 4:**
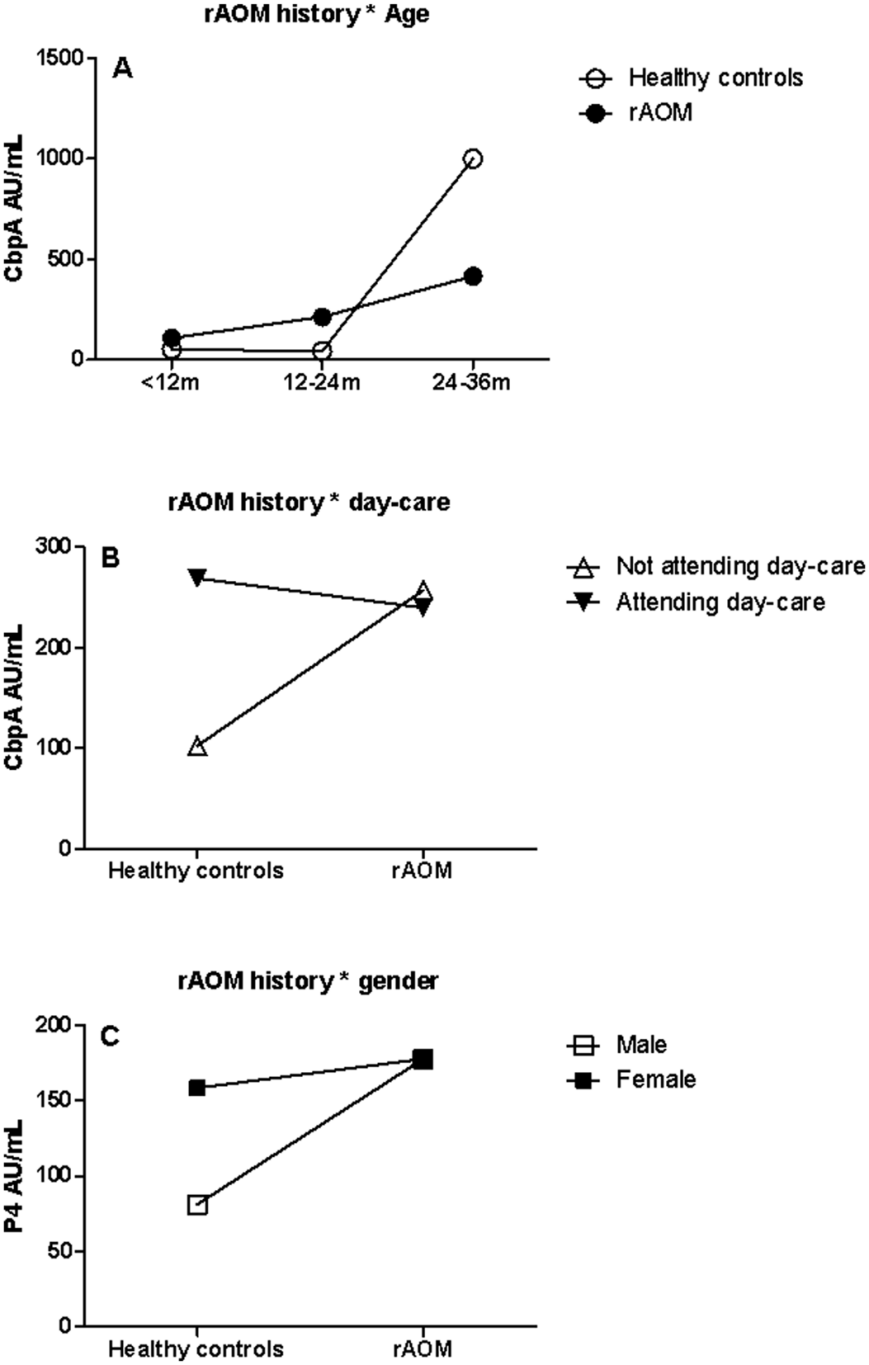
Interactive effects. Interactive effects calculated using general linear model of rAOM * age (panel A; p = 0.003) and rAOM * day-care attendance (panel B; p = 0.04) on geometric mean CbpA levels (AU/mL) and the effect of rAOM * gender on GMC of anti-P4 IgG levels (panel C; p = 0.05). *CbpA, choline binding protein A; P4, outer membrane protein 4.*

### Measurement of IgG Against Pneumococcal and NTHi Proteins using a Multiplex Bead Assay

Microspheres were incubated with bead activation buffer (1×PBS containing 5 mg/mL 1-ethyl-3-(-3dimethylaminopropyl)-carbodiimide hydrochloride and 5 mg/mL N-Hydroxysuccinimide) for 20 minutes. Recombinant purified proteins were then covalently conjugated to these activated fluorescent carboxylated microspheres using a two-step carbodiimide reaction. To assess assay specificity, a selection of serum samples were pre-incubated with a single recombinant protein prior to performing the multiplex assay, which should only affect the antibody measurement against that specific protein. However, we found that addition of PspA1 interfered with measurement of PspA2, CbpA, and Ply antibody levels and that pre-incubation of serum with PD interfered with measurement of P4 antibody levels. To prevent this cross-reactivity and ensure optimal assay specificity, test samples were measured using a 2-plex assay including microspheres coated with PspA1 and PD and a 5-plex assay including microspheres coated with PspA2, CbpA, Ply, P4, and P6. In each assay, pooled sera from adult volunteers were used as a reference and quality control and IgG-deficient serum (Sigma-Aldrich) was used as a negative control.

With this optimised assay, test serum samples were diluted 1∶250 in phosphate buffered saline containing 2% new born bovine serum and 0.05% Tween20 (All Sigma-Aldrich). Diluted samples (25 µL) were incubated with a mixture of 25 µL of the protein-coated microspheres for 30 minutes at room temperature. After washing (phosphate buffered saline, 0.05% Tween20), 100 µL of *R*-phycoerythrin conjugated anti-human IgG was added (Jackson ImmunoResearch Laboratories, Pennsylvania, USA), the mixture was incubated for 30 minutes at room temperature and washed again. The fluorescence of 100 beads within each specific bead region was measured on the BioPlex® 200 System (Biorad). Inter-assay variability was assessed by calculating the % CV of the mean fluorescence intensity of standard curve dilutions 4 and 7 over all assays which were in the range of 10–15%. Mean fluorescence intensity data was acquired electronically in real-time and analysed using Bio-plex Manager 5.0 software. Data in arbitrary units (AU/mL) were generated from a 5-PL standard curve of mean fluorescence intensity against the in-house reference human serum pool and plotted as the geometric mean concentration (GMC) with 95% confidence intervals (CI). Out of range values were repeated using appropriate higher or lower serum dilutions.

### Detection of *S. pneumoniae* and NTHi in Nasopharyngeal Swabs and Middle Ear Effusion

Nasopharyngeal swabs were examined for the presence of *S. pneumoniae* and NTHi using standard culture methods. PCR for detections of *S. pneumoniae* and *H. influenzae* was conducted on genomic DNA prepared from the middle ear effusion. Methods and results of these analyses have been described in detail elsewhere [20].

### Statistical Analyses

Host and environmental risk factors were compared between children with or without rAOM using Student’s *t* tests for continuous variables (age) and Pearson Chi-square analyses (p-value asymptotic significant 2-sided) for categorical variables (gender, day-care attendance, siblings, and PCV7 vaccine status). Antibody titers were expressed in Arbitrary units/mL (AU/mL) against a reference human serum pool and reported as adjusted antigen-specific geometric mean concentrations (GMCs) with 95% confidence interval (CI), correcting for confounding variables as appropriate. Adjusted GMCs were calculated using adjusted antibody concentrations that were computed in log values for the adjustment. The residual values that were derived in a linear regression model or general linear model with the corresponding antibody levels as dependent variable and the confounders as independent variables [23], [24] were used for the calculation of the adjusted antibody levels. To analyse differences in anti-pneumococcal (PspA1, PspA2, CbpA, and Ply) and anti-NTHi (P4, P6, and PD) antibody levels between children with or without rAOM linear regression correcting for age, gender and day-care attendance was used. To analyse the difference in antibody levels between children who were either colonised or not colonised with *S. pneumoniae* or NTHi linear regression correcting for age, gender and day-care attendance was used. These analyses were conducted within the group of children with a history of rAOM and within the group of healthy controls, separately. General linear model with Tukey honestly significant difference test was used to compare the difference in antibody levels in three different age groups (<12 months, 12–24 months, and 24–36 months of age) within the group of children with a history of rAOM and within the group of healthy children, after adjusting for gender and day-care attendance. General linear model was also employed to investigate the two-way interactions of rAOM with age (categorical variables), gender and day-care attendance. IBM SPSS Statistics 19 for Windows software package (IBM, New York, USA) was used for all statistical analyses and data were plotted using GraphPad Prism (Graphpad Software Inc, California, USA).

## Results

### Study Population

We enrolled 172 children with validated medical records meeting the study inclusion criteria of a history of at least 3 episodes of AOM in the first 3 years of life (median 7 AOM episodes) requiring the insertion of ventilation tubes. The healthy control group was comprised of 63 children without a history of ear disease. The mean age of the group of children with a history of rAOM was 21.1 months (range 7.3−36.0) and of controls 18.7 months (range 7.0–35.0; p = 0.06), with median ages of 20.6 months and 15.8 months, respectively. In the group of children with rAOM 61% were male and the control group consisted of 73% males (p = 0.09). None of the parents of children in the study self-identified as being Aboriginal or of Torres Strait Islander descent. In the rAOM group, 63% attended a day-care facility for more than 4 hrs/week, whereas this was 31% in the healthy control group (p<0.001). In both groups the percentage of children that had siblings was similar, being 70% in children with and 74% in children without a history of rAOM (p = 0.6). PCV7 vaccine coverage was also similar between children with (99% fully vaccinated) or without a history of rAOM (97% fully vaccinated; p = 0.3). *S. pneumoniae* and NTHi were more commonly isolated from the nasopharynx of children with a history of rAOM compared with healthy children (41% *vs* 27%; p = 0.04 for *S. pneumoniae* and 56% *vs* 21%; p<0.001 for NTHi).

### Serum IgG Levels to NTHi Proteins are Higher in Children with rAOM

In children with a history of rAOM the raw geometric mean serum IgG antibody concentration against pneumococcal proteins were 1.5−2.7 times higher than those in healthy children, however, these differences were not statistically significant using linear regression correcting for age, gender and day-care attendance (Figure 1). The group of children with rAOM had significantly higher (1.7−2.1-fold) GMCs against the NTHi proteins P4 (p<0.001), P6 (p = 0.01), and PD (p = 0.01) compared with the healthy control group (Figure 1) after adjusting for age, gender and day-care attendance.

### Serum IgG Levels According to AOM History and Nasopharyngeal Colonisation

We then investigated the effect of colonisation on antibody production in healthy children compared with children with a history of rAOM. Children with a history of rAOM and colonised with *S. pneumoniae* had 1.8−3.4 times higher antibody levels compared with cases that were not colonised (PspA1: p<0.001; PspA2: p = 0.003; CbpA: p<0.001; Ply: p = 0.003; Figure 2). Similarly, cases colonised with NTHi had significantly higher geometric mean IgG concentrations against P4 (p = 0.006), P6 (p = 0.001) and PD (p = 0.04) compared with cases that were non-carriers of NTHi (Figure 2). Healthy children that were colonised with *S. pneumoniae* had GMCs that were significantly higher against PspA1 (3.5 times higher, p = 0.008) and CbpA (4.4 times higher, p = 0.006), whereas the GMCs to PspA2 and Ply were not different in healthy controls carrying *S. pneumoniae* compared with those not colonised (Figure 2). The geometric mean antibody concentration against P4 (p = 0.02) and PD (p = 0.02) were significantly higher in healthy children colonised with NTHi compared with non-colonised healthy children (Figure 2).

### Serum IgG Levels do not Correlate with Detection of Pathogens in the Middle ear

Of the 134 children with rAOM that had middle ear effusion collected during surgery for the insertion of ventilation tubes, 8 were PCR positive for *S. pneumoniae* (6%) and 61 were PCR positive for NTHi (46%). There were no differences in geometric mean serum IgG levels against any of the pneumococcal and NTHi protein antigens between the children that did or did not have the respective pathogen in the middle ear (Table 1 and Table 2). The mean age, gender distribution and day-care attendance rates were not different between children that did or did not have *S. pneumoniae* and NTHi detected in the middle ear (data not shown). There were also no differences in geometric mean antibody concentrations against any of the protein antigens according to the number of AOM episodes experienced prior to entry in the study (data not shown).

### Serum IgG Levels to Bacterial Proteins Increase with Age

To investigate the effect of age on antibody levels, data in both groups of children, either with or without a history of rAOM, were analysed in 3 age-brackets: <12 months old, 12−24 months old, and 24−36 months old (Figure 3) using a general linear model after adjusting for gender and day-care attendance. In both groups antibody levels increased with age. This rise was most apparent for IgG against pneumococcal proteins, with a significant increase in children with a history of rAOM for PspA1, CbpA, and Ply (Figure 3). In healthy controls, antibody titers against the pneumococcal antigens remained low until 24 months of age, but were significantly higher in the 24−36 months old age group. There was a trend for higher antibody levels against all NTHi protein antigens with increasing age in both groups of children but this did not reach statistical significance. When comparing antibody levels against NTHi proteins between children with and without a history of rAOM within a specific age-bracket, antibody levels in children with rAOM were generally higher.

### The Interactive Effects of rAOM with Age, Gender and Day-care Attendance on Serum IgG Levels

To clarify the effects of rAOM on serum IgG levels we further investigated the interactive effects of a history of rAOM with age, gender and day-care attendance. For the 7 antibody levels, we identified three significant interactions: rAOM*age on CbpA (p = 0.003), rAOM*day-care attendance on CbpA (p = 0.04) and rAOM*gender on P4 (p = 0.05). Consistent with the findings in paragraph 3.5, CbpA levels increased with age and the linear pattern was more apparent in children with rAOM (Figure 4a). However, after two years CbpA levels increased significantly in controls. Children with a history of rAOM who did not attend day-care had significantly higher levels of antibodies against CbpA (p = 0.03). In contrast, a history of rAOM did not increase the levels of CbpA in children who did attend day-care (p = 0.79) (Figure 4b). In boys, a history of rAOM had significantly increased the levels of P4 antibody levels (p<0.001), however, rAOM did not increase the levels of P4 IgG in girls (p = 0.71) (Figure 4c).

## Discussion

In this large cross-sectional study of young children with a history of rAOM undergoing ventilation tube insertion and healthy age-matched controls, we have shown that children with a history of rAOM have significantly higher naturally acquired serum IgG levels to the NTHi proteins P4, P6, and PD. In addition, serum IgG levels to protein antigens were higher in children colonised with *S. pneumoniae* or NTHi, suggesting that serum IgG is induced by carriage. Together this may imply that the higher antibody levels in children with a history of rAOM are a marker of previous exposure rather than a measure of susceptibility to recurrent disease. To address this, longitudinal studies are required. Our findings of higher antigen-specific antibody responses in children with rAOM or children colonised with *S. pneumoniae* or NTHi are in accordance with several studies in children with AOM [10], [11], [12], [13], [14] or colonised with *S. pneumoniae* or NTHi [11], [14], [25], [26]. Our data contradict other studies, in which the observation of lower antigen-specific antibody levels in children with rAOM suggests that these antibodies are protective [15], [16], [17], [18], [19]. These conflicting findings may reflect differences in the timing of, or the response to colonisation in children with a history of rAOM compared with healthy children. One study showed that antibodies against CbpA and Ply were lower in children colonised with *S. pneumoniae*. Children in this study were undergoing surgery for adenoidal hypertrophy though and were not reported to suffer from rAOM, suggesting the aetiology of diseases is different [27]. Reduced antibody responses after colonisation may play a role in the progression of adenoidal hypertrophy, but may be less important in the development of rAOM.

It is important for these discrepancies to be resolved to improve our understanding of the aetiology of AOM, which could contribute to the development of diagnostics that would allow for the prediction of prognosis, early intervention and improved treatment options for children with AOM. Studies investigating pneumococcal and NTHi specific immune responses in children with rAOM should be compared cautiously though, especially when the aim is to draw general conclusions on the immune status of children with rAOM or the immunogenicity of vaccine candidate proteins in these children. Firstly, investigation of samples collected during or outside the acute phase of infection may lead to different results and conclusions. The high antibody levels in children with a history of rAOM between AOM episodes, as we show here, may reflect a booster-type of response induced by previous colonisation and/or disease, whereas during acute infection the antibody response in otitis-prone children may well lag behind. Two recent studies from the same investigators compared IgG levels to pneumococcal and NTHi proteins in serum all collected during an AOM episode from otitis-prone children, non otitis-prone children and children who failed to respond to antibiotic treatment [18], [19]. The authors concluded that otitis-prone children are immunologically hypo-responsive to pneumococcal and NTHi proteins. Outside the acute infection however, the antibody titers in otitis-prone children deemed to be similar to those in controls, but direct statistical analyses were not described. These studies rather analysed the kinetics of antibody responses, which actually showed higher and earlier, but flatter antibody responses in otitis-prone children. Care should be taken in generalising results from these smaller and clinically less distinct cohorts, as these results may not reflect the immune status of the majority of children with rAOM.

In addition it is crucial to assess age-matched cohorts with a strict, limited age-range when investigating immunity in children with rAOM, since antibody titres against pneumococcal and NTHi protein antigens increase with age as shown here and by others [11], [14], [19], [26], [28]. Data from studies with a wide age-range [27] or where otitis-prone and non otitis-prone groups are not age-matched [17] should be interpreted cautiously. The conclusions drawn from such studies that state that otitis-proneness is related to several immunological derangements seem to be too strong.

Moreover, antibody levels against a wide range of antigens should be tested before conclusion on the general immune response in children with rAOM are made, since antibody responses against antigens are very different, partly due to variation in expression patterns and function of specific proteins in bacterial strains and species. The country and era that samples were collected in also needs to be taken into account, since pneumococcal and NTHi carriage patterns, and therefore immune responses, can vary, for instance due to the use of pneumococcal conjugate vaccines.

Finally, there were interactions between rAOM and gender, age and daycare attendance on antibody levels. The effects of rAOM on antibody levels varied between sub-groups of children with different combinations of confounders. This further complicates the investigation on the relationship between rAOM and antibody responses in children.

Considering the conflicting data, the complex interactions identified in this study and conclusions regarding antibody responses in children with rAOM in literature, partly due to the limitations described above, there is a clear requirement for larger, well age-matched studies in which standardised clinical definitions of the rAOM phenotype are applied and samples are collected during the same phase of the AOM episode. In addition it will be important to investigate the timing and load of bacterial colonisation and/or disease, antibody levels, antibody functionality, mucosal immunity and cell mediated immune responses to bacterial otopathogens. These factors are likely to be interacting and therefore large longitudinal studies following children from early on in life are necessary to better understand the aetiology and pathogenesis of rAOM. Data from our large cross-sectional study shows that several pneumococcal and NTHi proteins are immunogenic in young children with a history of rAOM. While a subset of children with humoral immune-deficiencies may suffer from rAOM, our findings indicate that previous statements suggesting that all children with rAOM have impaired antibody responses should be reconsidered. The fact that otitis-prone children can mount antibody responses to proteins from common otopathogens implies that a vaccine including a combination of protein antigens may offer protection from initial colonisation and otitis media when used early in life. The specific mechanisms in which bacterial proteins induce immunity and potential protection against rAOM when used in protein based vaccines, requires further investigation.
